# Facilitated Peer Discussion for Promoting Better Resident Wellness in Anesthesia Trainees: Qualitative Program Evaluation

**DOI:** 10.2196/78575

**Published:** 2025-12-01

**Authors:** Miku Wake, Nicholas West, Jessica Luo, Nancy Wang, James D Taylor, Kyra Moura, Theresa Newlove, Zoë Brown

**Affiliations:** 1 Faculty of Science, University of British Columbia, Vancouver, BC, Canada; 2 Department of Anesthesia, BC Children's Hospital, T3-246, 4480 Oak St, Vancouver, BC, V6H 3V4, Canada, 1 604-875-2711; 3 Research Institute, BC Children's Hospital, Vancouver, BC, Canada; 4 Department of Anesthesiology, Pharmacology & Therapeutics, University of British Columbia, Vancouver, BC, Canada; 5 People Experience & Workplace Wellness, Provincial Health Services Authority, Vancouver, BC, Canada; 6 Department of Psychology, University of British Columbia, Vancouver, BC, Canada

**Keywords:** anesthesia, resident wellness, burnout, facilitated peer discussion, semistructured interview, survey

## Abstract

**Background:**

Anesthesia residents experience nonroutine clinical events during perioperative patient care, including workplace stressors or adverse incidents that may cause physical and emotional stress. These events can lead to burnout and negative mental health outcomes. Burnout and depression rates are lower when residents have adequate support systems within their workplace. Better resident wellness (BREW) Rounds are a weekly 1-hour peer discussion for anesthesia residents, facilitated by a registered psychologist at our institution. Although shown to improve residents’ well-being, a deeper understanding of the benefits of such programs may support their expansion to other residency programs.

**Objective:**

This study aimed to explore the benefits and most effective features of BREW Rounds to guide the development of similar programs at other institutions.

**Methods:**

Following research ethics board approval, we conducted a qualitative descriptive study based on semistructured interviews with anesthesia residents who had participated in one or more BREW sessions and with the main BREW Rounds facilitator. Topics of discussion included community building, belonging, mentorship, facilitation, discussion of nonclinical aspects, and removal of hierarchy. Interviews were conducted on videoconferencing software by researchers who were not involved in supervising or assessing the trainees. Audio recordings were auto-transcribed, deidentified, verified, and interpreted using thematic content analysis. Further perspectives on BREW Rounds were obtained from staff anesthesiologists through an anonymous online survey.

**Results:**

We interviewed 10 residents (6 junior, 3 senior, and 1 transition-to-practice) and 1 facilitator. Emerging themes included (1) access to a safe space free of judgment, allowing participants to be vulnerable about clinical or nonclinical aspects of their training, (2) building a sense of community in a fast-paced and often isolating environment, (3) providing opportunities for mentorship between junior and senior residents in a frequently changing colleague network, (4) the characteristics that create a “BREW culture”, such as behavior norms during sessions and staff respect for protected time, (5) the importance of a good facilitator from outside the anesthesia department, especially during smaller sessions, (6) expanding BREW Rounds to other institutions, and (7) areas for improvement for the current program. Sixteen anesthesiology staff survey responses were available for analysis: 12/16 (75%) anesthesiologists supported residents leaving their clinical duties early for BREW Rounds and 12/16 (75%) believed BREW Rounds benefitted residents’ well-being.

**Conclusions:**

This qualitative study confirms previous findings that BREW Rounds are beneficial to anesthesia training, improve the psychological wellness of residents, and may positively contribute to patient care. Program directors should recognize their potential positive impact on the learning environment, ensure that all staff and trainees understand the need to create protected time for this activity, consider partnering with wellness initiatives at the institutions in which residents are training, and endeavor to identify experienced and unbiased facilitators to moderate sessions.

## Introduction

Anesthesia residents experience nonroutine clinical events during perioperative patient care. These can include adverse events involving actual or potential patient harm, as well as nonsafety-related situations of physical and emotional stress. These occur alongside everyday workplace stressors such as work–life balance, social tension or isolation, and the emotional impact of adverse patient outcomes [1-4]. Although resident wellness and its factors are well recognized, anesthesia care has historically focused on preventing adverse events rather than creating the necessary systems to support physicians navigating conflict [5,6]. Furthermore, anesthesia tends to be a siloed department. Its residency partakes in a one-on-one system, where each trainee is paired with one staff member every day. Many struggle with finding social connection and community within their peer group, both of which play a vital role in supporting psychological well-being throughout training and into their careers as attending physicians [7].

The understanding surrounding resident burnout is still preliminary [8-10]. However, it has been reported that the risk of burnout and depression is lower when residents have adequate workplace resource availabilities, better work–life balance, and the required social support [11]. Reducing loneliness and enhancing community in the anesthesia department will improve mental health outcomes for trainees, which may benefit them personally, as well as translate to overall contributions in the clinical setting [7].

In response to findings from a survey of University of British Columbia (UBC) anesthesia residents [12], we implemented better resident wellness (BREW) Rounds at British Columbia Children’s Hospital (BCCH) in July 2021 for residents undergoing their pediatric anesthesia rotation. These facilitated peer discussions take place every Thursday from 3 to 4 PM at BCCH, away from the anesthesia department, with snacks provided. Residents are excused from their clinical duties early and given the option to attend. BREW Rounds are facilitated by a registered clinical psychologist, who is the institution’s People Experience & Workplace Wellness director and who has facilitated peer discussion groups with other health care teams. BREW Rounds provide residents with a safe space of facilitated peer discussion, with the goal to foster a healthy and psychologically positive professional environment. Conversations are confidential, open-ended, and participant-driven, and the program is strictly intended for anesthesia residents only. Attendance varies with call schedules and the number of residents on rotation; residents attend a median (IQR) of 2 (1-3) BREW sessions in a 4-week period, and there are typically 3‐4 residents in each session, ranging from 1 (rarely) to 7. Discussion topics typically include communication issues, health care team and patient interactions, and the impact of critical and other distressing events [13].

Residents provided positive feedback in pre- and postintervention surveys, with 96% finding BREW Rounds helpful and 88% experiencing a boost in their morale [13]. However, the surveys did not fully explain the perceived benefits or most effective features of BREW Rounds, and we concluded that further qualitative investigations were indicated. This study is intended to improve our understanding of the BREW program, determine the key features of the current approach, and identify opportunities for improvement, which may support the expansion of facilitated peer discussions to other residency programs and institutions.

## Methods

### Study Design

We conducted a qualitative descriptive study based on semistructured interviews with a sample of anesthesia residents who had attended BREW Rounds, as well as the main facilitator. The focus of the interviews was derived from themes that had been constructed from free-text comments made in feedback surveys completed by residents who had attended BREW Rounds previously [13]. Hence, we aimed to understand and explore themes related to bonding, mentorship, facilitation, opportunity for the discussion of nonclinical aspects of events, removal of hierarchy, and, in particular, to investigate if, how, and why BREW Rounds contributed to the concept of “community-building” [14]. Further, we conducted a brief online survey to gain the staff anesthesiologists’ perspectives on the BREW program. This manuscript follows the consolidated criteria for reporting qualitative research guidelines [15].

### Participants

#### Semistructured Interviews

Eligible participants for this study included the facilitators for BREW Rounds and current or recently graduated UBC anesthesia residents who had previously attended at least one BREW Round. Residents typically rotate through BCCH twice in residency, in 4-, 6-, or 8-week blocks, during their junior residency year 2 (R2) and their senior residency year 4 (R4); residents may also have a placement at BCCH during their transition-to-practice (TTP) at the end of residency year 5. The facilitator was recruited through email, and residents were recruited by either invitation to participate via email or a Research Electronic Data Capture (REDCap) survey [16] that they completed at the end of their rotation.

Participants were made aware that the study was being conducted to better understand BREW Rounds and that interviewers were not involved with their management, supervision, or assessment. Once an eligible participant expressed interest in the interview, their email address was added to the BREW interview electronic consent (eConsent) database on REDCap and an eConsent form was generated. Research staff coordinated a Zoom interview time and date via email.

#### Staff Survey

An invitation outlining the premise of the study and a link to a REDCap survey (see Multimedia Appendix 1) was created by the research team and sent to staff anesthesiologists at BCCH by the Principal Investigator (ZB) via a departmental email distribution list.

### Data Collection

#### Semistructured Interviews

The interviews followed a semistructured format with a preestablished interview script (see Multimedia Appendix 2). At the start of each interview, the lead interviewer introduced all parties and assured participants that there were no right or wrong answers, that none of the interviewers had experienced BREW Rounds firsthand, that the conversation was confidential, and that the interviewing investigators were not involved in managing, supervising, or assessing the residents or BREW Rounds facilitators. Data collection began after confirming their interest to participate and verbal confirmation of consent to record the interview. Interviews lasted approximately 45 minutes and were ended at the interviewers’ discretion that all topics had been covered and the interviewee had no further contributions. Each interview was conducted by 2 or 3 investigators from the Pediatric Anesthesia Research Team: N West (male, Clinical Research Coordinator, 13 y experience) conducted 9 interviews; JL (female, Clinical Research Assistant, 1 y experience) conducted 11 interviews; N Wang (female, Anesthesia Clinical Fellow) conducted 7 interviews; and MW (female, Clinical Research Assistant, 1 y experience) conducted 2 interviews.

The investigators had an existing working relationship with the BREW Rounds’ facilitator who participated in the study; N West had previously worked with the TTP resident on a quality improvement project; N Wang had worked in the same clinical setting as the resident participants. No other participants had any existing relationship with the investigators, although they may have encountered them during their placement at the hospital.

We used the Zoom software to automatically transcribe the recorded audio from the interviews. Two investigators (MW and JL) deidentified the participant’s Zoom ID, cleaned, and validated the transcript files with reference to the interview recording.

#### Staff Survey

After the initial invitation email was sent out, reminders were sent to staff through signal messages (two times) and emails (two times), and the survey was closed after 14 days once we felt that no further responses would be received.

### Data Analysis

#### Semistructured Interviews

Analysis followed a qualitative descriptive approach [17,18] based on thematic content analysis. Interview transcripts were analyzed using NVivo R1 (version 1.7.2 Lumivero) to construct themes using both a conventional content analysis method [19], in which initial coded themes were identified by one researcher before discussion with the team, and directed content analysis to confirm whether and how our data aligned with the preconceived theme of “community-building”; this process was conducted iteratively (see below). We aimed to continue recruitment until we reached sufficient information depth and breadth, and all interested groups were sufficiently represented, that is, we had achieved a balance of junior, senior, and facilitator participants.

MW first watched all interviews that they had not attended to become familiarized with the ideas discussed in each conversation. Using the auto-transcribed files from the Zoom software in conjunction with the interview recordings, the raw files were deidentified, formatted, and validated. The validated transcripts were read in-depth once more during initial thematic charting, in which main themes and subthemes were coded and refined based on the frequency, depth of discussion, and perceived importance of topics by MW. The updated themes were reviewed with N West, N Wang, and JL and finalized to best represent the captured concepts. The data were then recoded using the updated themes to improve its accuracy into each respective theme that was established.

The quotes used for this manuscript were chosen based on what was thought to best capture each finalized theme or subtheme, as well as keeping a balance of representing all interview participants’ ideas. Sample quotes associated with its description can be recognized as italic font, with credit to the deidentified participant type and/or number at the end. The quotes were preserved to their original transcription to the furthest extent. Finally, the themes, their descriptions, and representative quotes were verified with one of the resident interview participants and the BREW facilitator.

#### Staff Survey

Survey data were exported from REDCap to an Excel Microsoft (Redmond, WA) file. Likert-scale statement responses were grouped into five categories: strongly disagree, disagree, neutral, agree, strongly agree. Response choices for each statement were recorded as a frequency and a percentage. Survey comments were categorized based on their content.

### Ethical Considerations

#### Human Subject Research

This study was reviewed and approved by the UBC/Children’s and Women’s Health Center of British Columbia Ethics Board (H22-03516). The initial application with semistructured interviews was approved on January 25, 2023. An amendment was made to include the staff survey, which was approved on September 10, 2024.

#### Consent Procedure

Participants who were recruited and expressed interest in our project were sent a Research Ethics Board–approved invitation outlining the premise and aim of the study, as well as what participation entailed. Semistructured interview participants then signed a REDCap eConsent form prior to setting up any interview details.

Consent was implied for staff anesthesiologists who filled out the survey.

#### Privacy and Confidentiality

Interviews were not anonymous to the research team as they were conducted over Zoom, but transcripts were later deidentified. Any personal information provided was protected by the privacy law in British Columbia, the Freedom of Information and Protection of Privacy Act. We collected this information under section 26 (e) of Freedom of Information and Protection of Privacy Act.

Staff survey responses were anonymous to the research team unless participants included any identifiable information in their free-text responses.

#### Compensation

A CAD $50 (equivalent to approximately US $37) Starbucks gift card was sent to resident participants who joined the interview. There was no compensation for completing the staff survey.

## Results

### Semistructured Interviews

Following the expression of interest to participate in these interviews from the postrotation feedback survey sent to all residents or through word-of-mouth, 14 invitation emails were sent to residents; eConsent forms were sent to 12 residents who replied to the invitation, and consent was obtained from 10 of these residents. Follow-up emails were sent, but no further replies were received. Although we had reached sufficient information depth with the first 8 resident participants (6 junior and 2 senior), we elected to include an additional 2 senior residents to better represent that group and to interview the main BREW facilitator. Hence, our final sample included the BREW Rounds facilitator (female) and 10 anesthesia residents: 6 male and 4 female; 6 junior residents (R2), 3 senior residents (R4), and 1 TTP resident.

**Figure 1.**
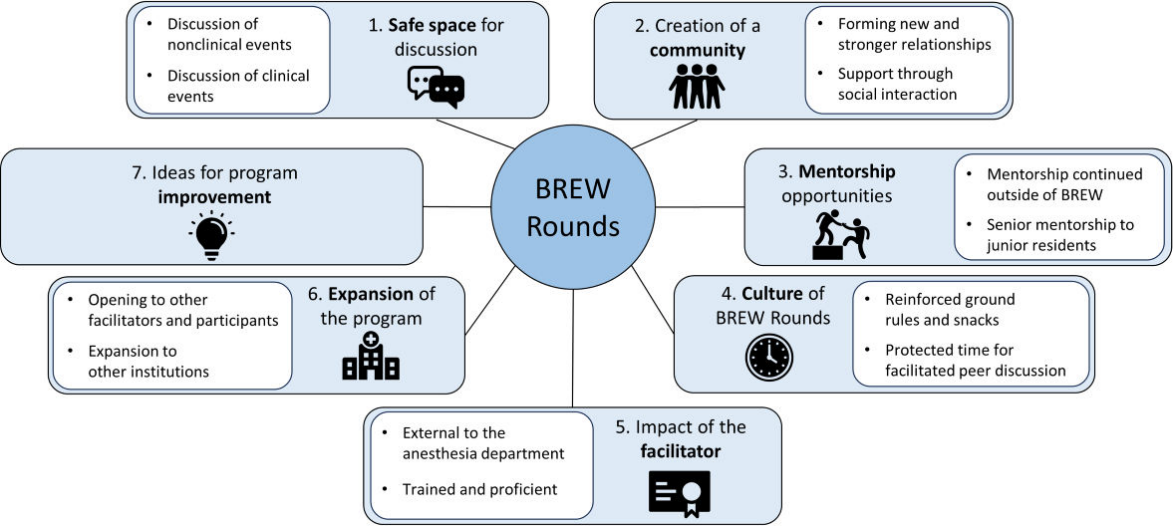
BREW Rounds interview themes. Visualization of the seven major themes and respective sub-themes constructed from semi-structured interviews. BREW: better resident wellness.

From the interviews, we constructed 7 major themes: (1) safe space, (2) community, (3) mentorship, (4) culture, (5) facilitation, (6) expansion, and (7) improvement. Within these major themes, several subthemes were also identified (Figure 1). Each theme and their respective subthemes are described in the following sections.

### Theme 1. Safe Space

This theme describes what BREW Rounds provided for the residents who attended them. Residents indicated that they were able to access a space where there was no judgment and were openly able to share their feelings in a confidential manner.

*I think that that’s one thing that you don’t get anywhere else in residency, just a safe space to complain.*

Participant 1 [R2]

Subthemes explored the content of what was talked about within BREW Rounds: discussions of both clinical events and nonclinical events. Examples of clinical events were anesthetic techniques or specific cases, while nonclinical events included complex interpersonal relationships between residents and staff or social topics such as their weekend plans. Some residents stated that though they were not in a clinical environment during BREW Rounds, they still felt that they were gaining clinical knowledge.

*When you talk about clinical things…we’re learning from each other about how best to give an anesthetic. That is definitely powerful because with your peers, it’s a lot easier to ask silly questions that you wouldn’t otherwise want to ask an attending.*

Participant 10 [R4]

*I think it’s a safe space where you can debrief, but also a nice space where you can walk out of that pretty busy rotation where you’re on call a lot and learning a lot.*

Participant 3 [R2]

Three residents mentioned that although BREW Rounds provided a safe space, that could be compromised by other attendees with a different attitude toward these sessions. For example, one junior resident expressed feelings of discomfort about some of the senior residents, and another interviewee said they had felt uncomfortable sharing challenging experiences during BREW rounds when the only other participant was very nonchalant. The facilitator also mentioned one incident where there were unsafe words said at a BREW round. Finally, one senior resident expressed concern that safety would be hard to maintain in a larger group (see Theme 6). However, residents who raised this point felt that they were able to distinguish who was safe from those who may not be.

*I think that the staff respect it, and in that sense it’s safe. I do think, though, that the people who are not safe people in our residency program have the ability to take that safe space and make it unsafe.*

Participant 5 [R4]

### Theme 2. Community

Resident interviewees also discussed the bonding and newfound camaraderie initiated by their participation in BREW Rounds, which continued beyond the sessions themselves and was deemed especially important in what they called a “siloed field.” The subthemes included the building of new relationships and the positive impact of these social interactions on their well-being.

*I think it builds a team –we don't get team lunches, so it’s like taking just a little bit of time to build that sense of team and get to know different people.*

Participant 3 [R2]

Through simple conversations with peers during BREW Rounds, they indicated that they were able to make connections that otherwise would have been hard to foster between residents in different years of anesthesia training.

In an environment where trainees work one-on-one with staff, residents noted the lack of interactions between themselves. When they did see each other, it was in the context of lectures and clinical practice, rather than casual conversation.

*I think that it’s sometimes nice to talk to people that you wouldn't otherwise talk to in that kind of context about unrelated things.*

Participant 8 [R4]

*You get to have these inter-year relationships, which I think is helpful and builds camaraderie within the residency.*

Participant 6 [R2]

### Theme 3. Mentorship

Several interviewees commented on the idea of mentorship growing out of BREW Rounds. Subthemes were the concept of senior–junior mentorship and that these relationships persisted beyond BREW Rounds. The main form of mentorship was between the juniors and seniors. Overlapping with the aspects of validation and social interactions that BREW Rounds provided, many juniors expressed their appreciation to seniors who shared their perspectives, learnings, and experiences.

*It was just nice to have…the validation of having people to vent with who actually know what’s going on…*

Participant 4 [R2]

*I've seen some really remarkable R4s be super proactive with no prompting, checking in with the R2s in the room.*

Participant 11 [facilitator]

From the other viewpoint, many senior residents stated that they welcomed the idea of passing down the support they got through BREW Rounds as a junior.

*As a senior, it was an opportunity to validate, normalize support, give pointers and tips, survival strategies, offer some signage to the juniors to help them and just listen to them and hear what they've been going through, and just be as non-judgmental as possible.*

Participant 5 [R4]

However, one participant discussed that when they were a junior resident, they were aware that those above them may take on roles of staff or evaluators before them. Having experienced this as a junior changed their perspective on how to behave as a senior resident in this situation.

*I found that when I was a junior, there were some seniors there that I was not as comfortable around and that sort of detracted from the experience a decent amount. Having had that experience, I tried to change that as much as I possibly could as a senior to make it as welcoming and open for the juniors.*

Participant 5 [R4]

Finally, multiple participants mentioned that mentorship was not limited to the weekly 1-hour sessions but extended into their clinical environment, whether it was in the current training site or at a different hospital.

*The senior residents that were on my block created a great culture…it wouldn’t feel awkward to approach them at any time and chat about what’s going on… I certainly approach them outside of formal BREW Rounds, and they were very helpful.*

Participant 1 [R2]

*I find that a lot of the senior residents…also provide that mentoring outside of BREW Rounds, even at other sites.*

Participant 2 [R2]

### Theme 4. Culture

BREW Rounds are now well established at BCCH and a “BREW culture” has emerged, which was described by interview participants. Subthemes related to the need to respect protected time for attending BREW Rounds and the ground rules—some stipulated, some that have developed organically—to set behavior norms within the sessions.

*I think the only expectation that we're told at the start of the session every time is this has to remain…respectful, confidential, and safe…it seems like people respect the confidentiality and the safety aspect of BREW Rounds.*

Participant 10 [R4]

Although not mandatory, residents said that most peers will take the opportunity to leave their clinical duties and attend BREW Rounds. The participants also found that staff usually had no issues letting them go early on BREW Round days. This aspect of protected time was important to residents.

*All the staff know that you have to send your resident off, no questions asked, so a lot of the staff that I was with–or all of them–were really good on sending you out by 3pm*

Participant 2 [R2]

Apart from protected time, the importance of snacks at BREW Rounds was also frequently mentioned. Residents stated that food was always a good incentive, making BREW Rounds feel more casual by getting to eat and chat with peers. One resident also mentioned the sense of security they felt from snacks being available, as a possible psychological support to help deal with some of the difficult issues discussed.

I*t’s nice to be able to briefly disengage from the conversation in your head, and I think that eating does that, so the conversation never gets too intense, especially if somebody has had a really emotional time that day.*

Participant 8 [R4]

### Theme 5. Facilitation

An aspect of BREW Rounds that contributed to its success was the presence of the facilitator and their approach to managing or guiding the conversation. All participants mentioned the ways in which the facilitator made their experience better. The facilitator plays an integral role in helping to organically continue conversations. Subthemes suggested that a trained and proficient facilitator is a key success factor for the program and the importance of them being external to the anesthesia department.

*I think a lot of the success of BREW Rounds is dependent on the facilitator. I think she does a very excellent job of engaging everyone in the room.*

Participant 7 [R2]

*She does a very good job of just letting us talk, which I think is something that’s very positive about how she does it.*

Participant 9 [TTP]

In addition, residents appreciated that the facilitation was external; the facilitators were in disciplines unrelated to anesthesia, and BREW Rounds took place in a separate area of the hospital, away from the operating rooms and their usual workplaces.

*The fact that it’s away from the department allows you to be a lot more honest.*

Participant 10 [R4]

The way in which the facilitator guides the progression of discussions was considered to be purposeful. The facilitator stated that it was important to remember that BREW Rounds were meant to alleviate stress and burnout for the residents, and the session was focused on having them speak and discuss with each other.

*I’m really talking from their perspective which is what I observe, because this isn’t a lecture. I’m not teaching them anything unless they ask for something specific.*

Participant 11 [facilitator]

### Theme 6. Expansion

The theme of expansion encompassed the subthemes of expanding BREW Rounds to other institutions and developing the existing BREW Rounds to include other facilitators or participants such as staff or fellows. When asked if participants thought BREW Rounds should be implemented at other institutions, most agreed that they would be beneficial.

*I think anesthesia especially is a specialty that would benefit the most from a weekly debrief session, so I think it should spread to all the other sites, and I think it'd be very useful.*

Participant 10 [R4]

*I think every single hospital and every anesthesia department in the country should have BREW Rounds for their residents.*

Participant 9 [TTP]

Many residents mentioned the introduction of a similar facilitated peer discussion group that has begun at another hospital site based on the positive impact BREW Rounds has made at BCCH. However, they also acknowledged the challenges in implementing this new program, with the biggest barrier being the lack of a facilitator like the one at BCCH.

*I think what we're struggling with right now is creating that magic space in the same way that there is at [BC] Children’s [Hospital]…we're having trouble creating that at [other hospital site] and because we don't have [the BCCH facilitator].*

Participant 7 [R2]

We also asked participants their thoughts on fellows or staff joining BREW Rounds. Most residents stated that fellows and staff, as individuals who are professionally a few steps ahead of them, may take away an element of the purpose of BREW.

*I think [including fellows] would change the dynamic and…I think it would still be safe, but…I think it would end up losing a lot of that open space where people would feel comfy saying things.*

Participant 4 [R2]

The 4 resident participants who commented on group size felt that the current number of attendees (typically 3‐4 per session) was reasonable but voiced concerns that larger numbers (8‐9 or more) could be a problem that may need to be addressed in the future.

*I can see that maybe when I come back as an R4, the cohort will be even bigger… when the cohort gets to like above 10 just because then it tends to be that, like the louder voices will speak, and then the more quiet people will kind of like, sit and listen, which is like fine sometimes, but I do think in like a more cozy group it’s like easier to feel safer, I think.*

Participant 6 [R2]

### Theme 7. Improvement

Finally, we recorded suggestions for improving BREW Rounds that came up in our interviews. The main suggestions from the residents included keeping group sizes small and continuing to create a relaxed and safe space.

*I think maybe as the program expands, it'll be harder because there’s going to be tons of co-residents, and maybe you're less likely to feel comfortable and safe enough to share your experiences with a bigger group of people.*

Participant 10 [R4]

One resident commented on how their experience in BREW Rounds was also dependent on the willingness of other attendees to participate actively.

*Maybe something to think about for the future of BREW Rounds is, how is everyone feeling? Are we in a space where people are going to be receptive and open to those discussions?*

Participant 3 [R2]

On the contrary, some residents stated that they would keep BREW Rounds as it currently is.

*I was trying to think earlier today about what I could say about BREW Rounds without [making it] sound like I was so positive, but I couldn't think of any bad thing about it.*

Participant 9 [TTP]

Comments about how to best ensure all people were safe to open up to was another discussion point. From the facilitator’s perspective, creating a safe space for individuals to connect can result in, albeit rare, interactions that require intervention in the moment. For example, an instance in which a trainee mistook it as a place to raise unfiltered comments about another trainee who was present.

*Safe doesn’t mean you get to harshly and unfairly criticize people in the room.*

Participant 11 [facilitator]

The facilitator was also aware of previous feedback from an alumna stating that they sometimes struggled with the negativity that BREW Rounds can bring, if complaints are all that is talked about. The facilitators state they are always working to improve the complex aspects of BREW Rounds.

*That was a lesson learned…. although it is a safe space to share concerns and complaints, we have a consistent structured wrap up and rounding out of the session with perspective-taking and the group sharing what they have been grateful for over the week or what they are looking forward to in the next week….*

Participant 11 [facilitator]

### Staff Survey

**Figure 2.**
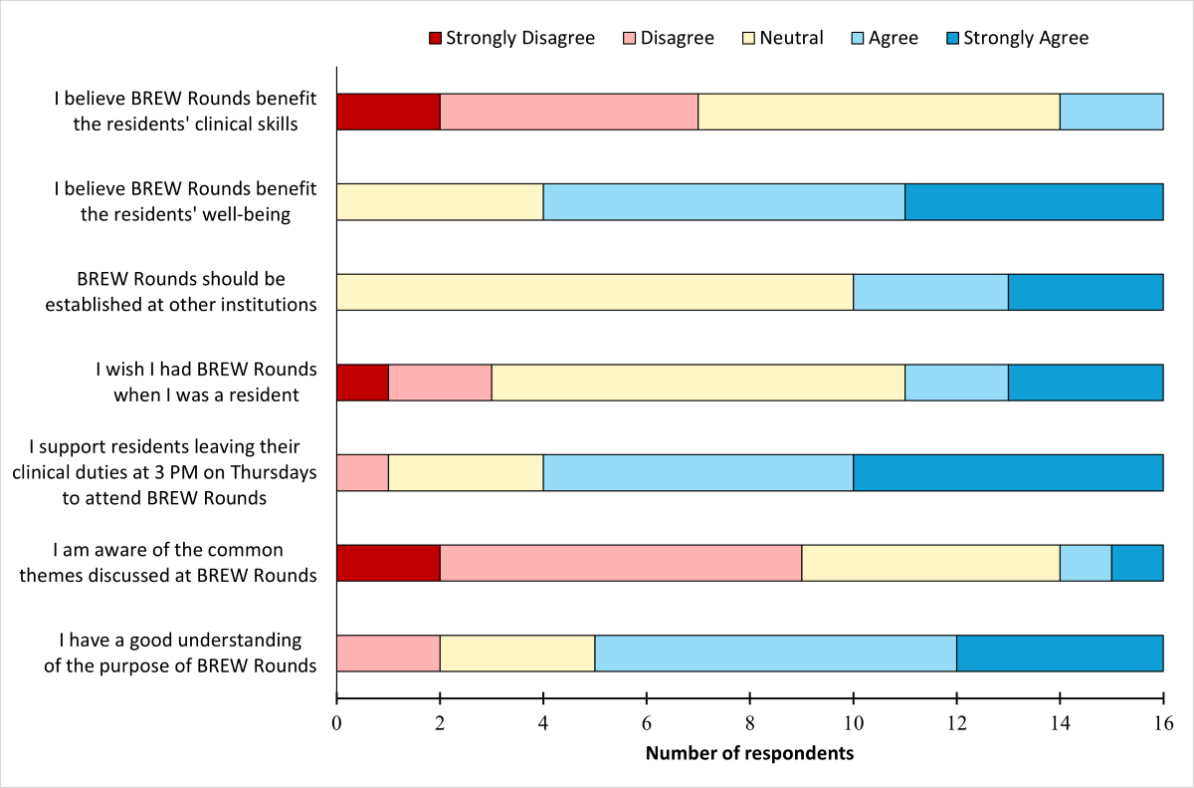
Staff survey results. Likert-scale responses to statements in the staff anesthesiologist survey. BREW: better resident wellness.

Sixteen out of 28 (57%) anesthesiologists completed the anonymous staff survey, excluding those who were on vacation leave at the time of survey distribution. Nine respondents also left free-text opinions on BREW Rounds. Overall, 12/16 (75%) staff (strongly) agreed that BREW Rounds were a benefit to the residents’ well-being (Figure 2). More than half (11/16, 69%) (strongly) agreed they had a good understanding of the purpose of BREW Rounds, and most (9/16, 56%) were not aware of the common themes discussed within the program.

Only a minority (2/16, 13%) of staff (strongly) believed BREW Rounds benefitted the residents’ clinical skills, and some staff members left comments regarding their concern toward loss of clinical time as a result of residents leaving early for BREW Rounds. On the other hand, others acknowledged the importance of resident wellness in exchange for an early dismissal.

*The requirement to lose potential clinical experience to attend is a shame.*

[Respondent 1]

*I am not sure if cases get discussed at BREW Rounds specifically so am not sure if the clinical aspect of helping each other with cases is there, but to me this is not the focus.*

[Respondent 6]

However, a majority (12/16, 75%) (strongly) supported residents leaving their clinical duties early every Thursday to attend BREW Rounds.

*I certainly don’t think that leaving at 15:00 for them once per week has a negative impact on patient care or on other aspects of resident training, and the residents seem to appreciate them.*

[Respondent 11]

## Discussion

### Principal Results

In this study, we developed a more refined understanding of the benefits of BREW Rounds. Themes from semistructured interviews with residents and the main BREW facilitator included (1) access to a safe space allowing vulnerability about clinical or nonclinical aspects of their training, (2) building a sense of community between residents in a fast-paced environment with a frequently changing colleague network, (3) mentorship opportunities between junior and senior residents, which can extend beyond the BREW sessions, (4) discussions of what characteristics create a “BREW culture,” such as the protected time designated for BREW Rounds every week, behavior norms, and provision of snacks, (5) the importance of having a good facilitator from outside the anesthesia department, especially during smaller sessions, (6) the potential for expansion of BREW Rounds to other institutions or expanding the current program to include additional participants or facilitators during some sessions, and (7) areas for improvement of the current program. The staff survey revealed that anesthesiologists supported residents leaving their clinical duties early for BREW Rounds, with 12/16 (75%) believing that BREW Rounds benefitted residents’ well-being.

### Comparison With Prior Work

#### Overview

These highlight the importance of including structured wellness programs, such as facilitated peer discussions into resident curriculums, which accords with other evaluations of similar programs within anesthesiology and other specialties [14,20-23]. We compared each of the main themes that we constructed from our interview transcripts with prior research.

#### Safe Space

Residents who have participated in peer discussion groups aimed at improving wellness appreciated being able to share their experiences and stressors, as well as hearing the challenges of others [14]. One of our interviewed residents voiced the concern of being vulnerable to seniors, and two others expressed anxiety around feeling “unsafe” in different situations during BREW Rounds. Creating a safe environment in which participants feel comfortable to share their experiences is essential and may be harder to achieve with a mix of junior and senior participants. This idea was well developed in a qualitative exploration of a peer discussion group for an emergency medicine residency program in California [21]; the investigators identified subthemes related to the “more experienced other,” with junior members being encouraged to share their own experiences after seeing their senior colleagues expressing vulnerability and finding comfort in knowing that senior colleagues had encountered similar challenges during their early training.

Staff from previous studies also show similar attitudes toward residency peer discussion programs. Program directors who responded to a survey by Chakravarti et al [20] recognized the importance of resident well-being and agreed that physician and resident wellness directly affects patient care. Although surveyed staff in our department did not think residents’ clinical skills would be improved by attending BREW Rounds, some of the residents we interviewed expressed an increase in their clinical knowledge from topics discussed during the sessions. Furthermore, a previous survey of BREW participants reported that 70% thought the program benefitted their clinical care [13]. While most staff understood the purpose of BREW Rounds, they were not aware of the content of discussions because of the agreed-upon confidential nature of the rounds.

#### Community

The space created for residents in BREW Rounds may also be contributing to the feelings of camaraderie and interconnectedness within their peer group. The sense of community gained by our resident participants from BREW Rounds was echoed in a study at the West Virginia School of Medicine, in which residents expressed the importance of social connectedness between themselves and their peers [22], which may be a particularly relevant issue for anesthesiology residents [7]. Similarly, Jain et al [21] found that residents who participated in a peer discussion group felt less isolated and felt a stronger sense of belonging within their cohort.

#### Mentorship

Existing research suggests that residents believe mentorship is a key factor in supporting their well-being [20]. Trainees who are mentored have increased achievements and satisfaction in their career overall [24,25]. We found that as a part of our initial goal of improving resident well-being through BREW Rounds, many residents appreciated the mentorship opportunities they obtained from the weekly sessions. In a 3-year curriculum plan aimed to improve wellness in anesthesia residency, Thornton et al [14] found that the most common positive feedback from residents was the small-group discussions, particularly with seniors and faculty. Furthermore, another study found that residents viewed the peer support program in the emergency medicine program to be more of a mentorship opportunity rather than a solution to burnout [21].

#### Culture

Residents from the University of Michigan partaking in a similar program stated that having the choice for level of participation increased their comfort and was appreciated [23]. Although it is mandatory for the residents to be let out from their clinical duties at 3 PM once a week, residents at BCCH are not obligated to attend BREW Rounds. The University of Michigan researchers acknowledged that lack of time spent for self-care and free time can contribute to burnout [23]. This highlights the importance of having protected time for BREW Rounds, combined with optional attendance, so that residents are free to attend if they wish to do so. All participants who were interviewed had positive associations and feedback for the snacks, which seemed to contribute greatly to the success of BREW Rounds. Residents from other studies also mentioned the importance of food as an incentive for engagement and to improve overall well-being [21-23].

#### Facilitation

Although Jain et al [21] evaluated a program with alumni as facilitators instead of an external, trained professional, similar themes of comfort, sense of belonging, and vulnerability were identified. Compared to the residents of our study who discussed the importance of having an external facilitator, their residents preferred talking to those with a similar background, stating they were able to understand the niche hardships they experience. Although not trained in anesthesia, our BREW facilitators have worked in the hospital setting for many years and have a high level of exposure to issues experienced by different specialists during their clinical care. Furthermore, in line with our facilitator’s interview, the primary responsibility of the facilitator is to keep the conversation safe and contribute perspective to the issues and concerns raised [21].

#### Expansion

When we asked residents what they thought of expanding the BREW Rounds to fellows, or even staff anesthesiologists, as participants or facilitators, most thought that including those who are higher on the career ladder would take away from the safe space that BREW Rounds currently affords. On the contrary, other researchers have reported that having alumni and staff join peer discussions can be seen more positively by the residents [14,21]. Most residents and some staff agreed that BREW Rounds, in the same format or similar, should be expanded to other institutions. While the initial motivation for BREW Rounds was based on the idea that a pediatric anesthesia rotation was potentially more stressful for trainees than other anesthesia environments, this idea was not borne out by the comments of our participants, who felt that other settings have their own challenges and would benefit from the BREW approach.

Four residents we interviewed mentioned a concern about the impact of growing cohort size, stating that it may be harder for everyone to have a chance to speak up at BREW Rounds. The ideal number of participants in BREW Rounds may be different for each individual. However, further consideration may be required in larger residency programs; participants deemed our typical BREW session group size (typically 3‐4) to be adequate or even optimal but had concerns that benefit would be lost for some participants in larger groups of 8‐10 or more. A descriptive analysis of free-text comments in the feedback obtained from over 500 obstetrics and gynecology residents following the implementation of a wellness curriculum in the United States suggested that small group size was considered important to facilitate engagement [26]. A recent qualitative study of resident peer reflection sessions in the Netherlands determined that 5‐7 was an optimal group size to ensure both depth of experience and safety for participants [27], which is consistent with our findings.

#### Improvement

Previous research highlights the negativity that can sometimes arise during peer discussions in the clinical setting [21]. This was also mentioned in our interviews, notably by the facilitator, who had received this feedback directly during the first year of BREW Rounds. Residents seemed to prefer informal peer discussion like BREW Rounds compared to formalized career improvement lessons surrounding topics such as conflict management, leadership, and communication [14].

There were discrepancies between resident interview comments and staff survey responses. In particular, some residents felt strongly that BREW Rounds had a positive impact on their clinical skills (see Theme 1), echoing a finding from our previous survey, which demonstrated reduced self-efficacy concerns following the BREW intervention, with 70% of participants reporting that their clinical care had benefitted [13]. On the other hand, in the present survey, only 2/16 (13%) staff believed BREW Rounds benefitted residents’ clinical skills. This may be partly due to different interpretations of “clinical skills” (activities involved in “hands-on” patient care vs broader professional capabilities, such as patient communication, leadership, and team management) but may also be due to the confidentiality surrounding BREW Rounds, which means that staff may be unaware that BREW topics include clinical scenarios and peer-to-peer teaching. Furthermore, staff may not have considered the strong evidence for an association between clinician well-being and clinical performance [28]. Educating staff on the general themes commonly discussed at BREW Rounds and the correlation between well-being and the development of clinical or professional skills may help to achieve departmental support. Our BREW facilitator recently presented at our departmental rounds on this topic. Ongoing outreach on the goals and content of the program, while maintaining confidentiality of individual conversations, could strengthen buy-in from interested parties and ensure its continuation.

### Limitations

Our study has some limitations. First, we had a small sample of interview and survey participants from a single institution. However, information sufficiency was reached, and we included a wide range of both junior and senior residents, as well as the BREW facilitator for the interviews. The staff survey was completed by 57% of department staff who were not involved in the study, with almost a third of staff on vacation during the time the survey was distributed. Second, although the offer to join an interview was given to all residents prior to their participation, residents were recruited on a volunteer basis or active recruitment, rather than randomly selected from the resident body. This may have resulted in dialogue with stronger opinions than the overall average, or a bias toward the benefit of BREW Rounds. Furthermore, residents may have had recall bias, as some were interviewed some months after their last attended BREW Rounds. Further investigations should explore the opinions from randomly selected residents, as soon as possible after their participation. Third, beyond gender and residency year, we did not collect ethnic, sociocultural, or other identity characteristics of our participants so as to ensure anonymity. Consequently, we cannot comment on the representativeness of our sample. Further research may be warranted to explore the BREW experiences of different identity groups, which may suggest different approaches to improve engagement and the potential benefit of the program.

In addition, BREW Rounds may not have the same number of participants every time, including the ratio of junior and senior residents. Not all of our participants attended the same number of BREW Rounds. This may have resulted in differences in the experience for each resident but likely broadly reflects the varying participation in BREW sessions. We did not send the transcripts back to all participants to have them confirm that their ideas were accurately reflected. However, the four interviewers, the BREW Rounds facilitator, and one resident participant all verified that the themes as described accurately represented the interview discussions. Finally, researchers may have had a natural unconscious bias toward the benefit of BREW Rounds in the interviews, which may have caused residents to respond in a certain way. Future studies should consider having external, impartial staff conduct interviews.

### Future Directions

BREW Rounds were first developed at a pediatric hospital with the consideration for the uniquely challenging aspects of providing pediatric anesthetic care. However, the themes that have emerged from the interviews are not exclusive to pediatric anesthesia and are likely still relevant across the range of anesthesia care at most hospitals that residents rotate through. It has yet to be seen whether these support rounds can provide benefit in other hospitals serving different patient populations. In the future, with the help of BREW Rounds alumni, we hope to see facilitated peer support rounds instituted and studied at other institutions in the province.

Further, as was apparent with the theme of “Facilitator,” the success of BREW Rounds is somewhat dependent on the facilitator available to guide the sessions. With the development of BREW Rounds at other hospitals, it will be pertinent to examine how different facilitators with various backgrounds add to or detract from the success of the support rounds. This will guide the generalizability of peer support rounds into other hospitals that may have different personnel resources available to facilitate the rounds.

We hope that this paper provides a framework for other hospitals, medical specialties, and residency programs to model their own support rounds. By prioritizing the themes constructed through the analysis of the interviews, an institution could create and expand on the idea of BREW Rounds at their own site.

### Conclusions

Through this study, we have confirmed that BREW Rounds are beneficial to anesthesia trainees and contribute to promoting overall resident well-being. Benefits of this program include having a chance to reflect on the emotional impact weighed on a trainee, better personal coping outcomes through “shared experiences,” increased team morale, and discussion of strategies to further enhance psychological safety in the hospital setting. This may, in turn, translate to improved patient care and clinical practice of future staff anesthesiologists. Program directors should understand the proper support systems that could be set in place to improve the well-being of physicians but also improve patient outcomes. This may include partnership development of wellness programs or partnerships with other initiatives to help combat resident burnout.

## Supplementary material

10.2196/78575 Multimedia Appendix 1 Staff survey.

10.2196/78575 Multimedia Appendix 2 Better resident wellness (BREW) Rounds interview script.

## Abbreviations

BCCH: British Columbia Children’s Hospital
BREW: better resident wellness
eConsent: electronic consent
R2: Resident, year 2
R4: Resident, year 4
REDCap: Research Electronic Data Capture
TTP: transition-to-practice resident
UBC: University of British Columbia
