# Supplementary material for: Facilitated Peer Discussion for Promoting Better Resident Wellness in Anesthesia Trainees: Qualitative Program Evaluation
Source: JMIR Perioper Med. 2025 Dec 1;8:e78575. doi: 10.2196/78575 (PMC12670191; doi:10.2196/78575)
Supplement: Multimedia Appendix 1 [file periop-v8-e78575-s001.pdf]

Staff Survey

A A A

Please complete the survey below.

Thank you!

Thank you for taking the time to participate in this survey.

The survey responses are confidential unless you include identifying information in your free-text responses. Your opinions and responses are protected by our privacy law in BC.

Please select the option that most accurately represents your opinion:

	Strongly disagree	Disagree	Neutral	Agree	Strongly agree
1) **I have a good understanding of the purpose of BREW Rounds** * must provide value	○	○	○	○	○
2) **I am aware of the common themes discussed at BREW Rounds** * must provide value	○	○	○	○	○
3) **I support residents leaving their clinical duties at 3pm on Thursdays to attend BREW Rounds** * must provide value	○	○	○	○	○
4) **I wish I had BREW Rounds when I was a resident** * must provide value	○	○	○	○	○
5) **BREW Rounds should be established at other institutions** * must provide value	○	○	○	○	○
6) **I believe BREW Rounds benefit the residents' well-being** * must provide value	○	○	○	○	○
7) **I believe BREW Rounds benefit the residents' clinical skills** * must provide value	○	○	○	○	○

8) **Any final comments:**

Submit

Powered by REDCap
