# Supplementary material for: Facilitated Peer Discussion for Promoting Better Resident Wellness in Anesthesia Trainees: Qualitative Program Evaluation
Source: JMIR Perioper Med. 2025 Dec 1;8:e78575. doi: 10.2196/78575 (PMC12670191; doi:10.2196/78575)
Supplement: Multimedia Appendix 2 [file periop-v8-e78575-s002.pdf]

BREW Rounds Interview Script

Introduction

Tell me about your experience participating in these sessions?

- How many BREW rounds did you attend?
- What was the most helpful aspect of the BREW rounds?
- What would you recommend to improve the BREW rounds?

What do you think you would miss out on if you did not attend these sessions?

I'm curious to find out about how you managed in the past with difficult scenarios. Previously when an incident has occurred, what resources would you have used?

Are clinical situations the main topic in BREW rounds? What other topics do you discuss or wish you could discuss. Have you had any other discussions of difficult situations in previous residency experiences?

Building community

1. **Removing hierarchy of residency**
    - How do you feel discussing issues with more junior or senior residents listening?
    - Tell me your experiences of hearing other residents discussing events that have affected them

2. **Belonging**
    - Do you think the support you provide each other evolves outside of the sessions? Have you reached out to someone in the resident group as a result of these sessions?
    - How have these sessions shaped your relationships with other residents?
    - What is meaningful to you that develops as a result of these sessions?
    - Why do you talk to some residents and not others, why?
    - Do you have other opportunities for similar sessions other than BREW rounds during residency?

3. **Mentoring**
    - As a junior / senior resident – has being a part of BREW rounds changed how you perceive / treat those who are junior / senior to you?

4. **Allowing a discussion of the non-clinical aspects of events**
    - We understand that conversations are guided to not dwell on the clinical aspects of a case. Do you find it valuable to debrief the non-clinical aspects of an event?
    - Do conversations occur that can divide the group, please describe?
    - Do conversations occur that unite the group, tell me more?

5. **Facilitation**
    - What does <the facilitator> add to the sessions?
    - Could you have your own sessions without a facilitator?
